# Supplementary material for: Clinical profile and hospital outcomes of children with Down syndrome diagnosed with congenital heart disease in a developing country: A retrospective study
Source: PLoS One. 2026 Apr 6;21(4):e0345457. doi: 10.1371/journal.pone.0345457 (PMC13052895; doi:10.1371/journal.pone.0345457)
Supplement: Attachment — Submitted filename: RESPONSE TO REVIEWERS-DOWN SYNDROME.docx [file pone.0345457.s003.docx]

RESPONSE MATRIX TO EDITOR AND REVIEWERS’ COMMENTS

**TITLE: CLINICAL PROFILE AND HOSPITAL OUTCOMES OF CHILDREN WITH DOWN SYNDROME DIAGNOSED WITH CONGENITAL HEART DISEASE IN A DEVELOPING COUNTRY. A RETROSPECTIVE STUDY**

| **COMMENTS** | **RESPONSE** | **CHANGES INSERTED IN TEXT ON PAGE NUMBER** |
| --- | --- | --- |
| The outline of the aim and objectives is poorly worded.  The presentation of the data is adequate in the results and the discussion section but the abstract is poorly outlined.  There are colloquialisms involved in the discussion that are unnecessary e.g. "at the other end of the pyramid"  Why are the mothers' ages described in median and the fathers in mean format.  There could be more referencing in the discussion portions, there are many assertions that appear to be congruent but they could be strengthened by seeking references.  The references are Vancouver style but there are at least 5 references that are in the incorrect format.  The Ethics clearance is current but it is not clear when it was initially sought. Is the ethics certificate an extension or only sought when the manuscript was submitted. In short, is ethics not sought before conduct of the research? | Thank you for your comment. We have made the necessary revisions.  Changes have been made, thank you  Thank you for the comment, the language has been revised.  The description of the parents ’ages was made according to the distribution of the data during analysis process; hence, measure of central tendency was used as suits for meaningful interpretation of the data, thank you  The revisions have been made in accordance with the suggested comments. Thank you!  Corrections have been made, thank you  Although the study had a retrospective design, ethical clearance was obtained before its commencement to access patient medical records and address various research ethical concerns. The approval number (Ref. No. AB.123/307/01K/39) was included in the manuscript after receiving Institutional Review Board (IRB) approval in June 2025. | **2**  **2**  **6**  **6**  **8,9** |
