# Supplementary material for: Clinical profile and hospital outcomes of children with Down syndrome diagnosed with congenital heart disease in a developing country: A retrospective study
Source: PLoS One. 2026 Apr 6;21(4):e0345457. doi: 10.1371/journal.pone.0345457 (PMC13052895; doi:10.1371/journal.pone.0345457)
Supplement: Attachment — Submitted filename: RESPONSE TO EDITORS COMMENTS.docx [file pone.0345457.s004.docx]

RESPONSE MATRIX TO EDITOR’S COMMENTS

**TITLE: CLINICAL PROFILE AND HOSPITAL OUTCOMES OF CHILDREN WITH DOWN SYNDROME DIAGNOSED WITH CONGENITAL HEART DISEASE IN A DEVELOPING COUNTRY. A RETROSPECTIVE STUDY**

| Additional Editor Comments | | |
| --- | --- | --- |
| **COMMENTS** | **RESPONSE** | **CHANGES INSERTED IN TEXT ON PAGE NUMBER** |
| The authors opined that "The goal is to assist healthcare providers and policymakers in improving care for these children.", however, there are no lessons to be drawn from this study. There is very limited data and result in just one table. There was no attempt to find any associations or inferences to the outcomes reported.  The Authors are therefore adviced to enhance the manuscript by 1. Reporting on echocardiographic details of cardiac lesions if any  2. Risk factors of adverse outcome    3. Further edit the manuscript for consistency in reporting as shoen in the abstract | Thank you for the comments.  In regard to lesson learnt from this study, at our centre, a significant number of children with congenital heart disease present late, with Down syndrome being at high risk of developing pulmonary hypertension, late presentation limit further surgical options. This study demonstrates a significant number of the enrolled children were first diagnosed beyond 5 months of age, which increases the odds of these children, either having delayed surgical repair or not fit for surgical intervention especially in resource limited settings. For example, surgical intervention for AVSD, a common lesion, is indicated below 6 months of age, preferably 3 months. There is also a figure included for the outcomes (mortality and length of hospital stay)  Thank you for the comment, ECHO was performed for each participant as per standard of care, however, our aim was to describe the commonest type of cardiac lesions among children with DS in comparison with previous studies. Hence, we reported the type of the cardiac lesion from the echocardiographic findings  In this study, we observed the small number of study participants because of the nature of the disease, which limited further statistical analysis as per study design. We also mentioned it as one of the limitation of this study.  We have made the necessary revisions accordingly. Thank you! | **2,4,5,6** |
